# Connecting EEG signal decomposition and response selection processes using the theory of event coding framework

**DOI:** 10.1002/hbm.24983

**Published:** 2020-03-09

**Authors:** Adam Takacs, Nicolas Zink, Nicole Wolff, Alexander Münchau, Moritz Mückschel, Christian Beste

**Affiliations:** ^1^ Cognitive Neurophysiology, Department of Child and Adolescent Psychiatry, Faculty of Medicine Technische Universitat Dresden Dresden Germany; ^2^ Institute of Neurogenetics University of Lübeck Lübeck Germany

**Keywords:** cognitive control, EEG, signal decomposition, small‐world network, theory of event coding, theta

## Abstract

The neurophysiological mechanisms underlying the integration of perception and action are an important topic in cognitive neuroscience. Yet, connections between neurophysiology and cognitive theoretical frameworks have rarely been established. The theory of event coding (TEC) details how perceptions and actions are associated (bound) in a common representational domain (the “event file”), but the neurophysiological mechanisms underlying these processes are hardly understood. We used complementary neurophysiological methods to examine the neurophysiology of event file processing (i.e., event‐related potentials [ERPs], temporal EEG signal decomposition, EEG source localization, time‐frequency decomposition, EEG network analysis). We show that the P3 ERP component and activity modulations in inferior parietal regions (BA40) reflect event file binding processes. The relevance of this parietal region is corroborated by source localization of temporally decomposed EEG data. We also show that temporal EEG signal decomposition reveals a pattern of results suggesting that event file processes can be dissociated from pure stimulus and response‐related processes in the EEG signal. Importantly, it is also documented that event file binding processes are reflected by modulations in the network architecture of theta frequency band activity. That is, when stimulus–response bindings in event files hamper response selection this was associated with a less efficient theta network organization. A more efficient organization was evident when stimulus–response binding in event files facilitated response selection. Small‐world network measures seem to reflect event file processing. The results show how cognitive‐theoretical assumptions of TEC can directly be mapped to the neurophysiology of response selection.

## 1 INTRODUCTION

A major topic in contemporary cognitive neuroscience research is the analysis of action control and response selection processes. From a cognitive theoretical point of view, many different frameworks have been proposed to explain action selection processes. One important framework is the “theory of event coding (TEC)” (Hommel, 2009; Hommel, Müsseler, Aschersleben, & Prinz, 2001), which explains how perceptions and actions (responses) are represented and how perceptions translate into appropriate responses. A central aspect in this “common coding” framework (Hommel et al., 2001) is that perceived external events (i.e., stimuli) and motor responses (actions) are represented by their features within a common format—the so‐called event file (Hommel, 2009). Stimuli are coded by features, such as their shape, color, or spatial position. Likewise, actions/responses are represented by features detailing the precise response, for example, which finger from which hand has to be activated. Stimulus features are stored in a so‐called “object file,” while features detailing the response are stored in a so‐called “action file.” The above‐mentioned event file comprises the object file and the action file and establishes associations (bindings) between each stimulus feature and each response feature (Hommel, 1998). Thus, an event file resembles a network of stimulus and response feature bindings (Hommel, 2011). A consequence of these network characteristics is that the entire event file can be (re‐)activated once a single feature of a stimulus, or a response, is (re‐)encountered (Hommel, 2011) and hence triggers the associated/bound response, or reevokes the respective stimulus features (Hommel, 2005). This “modus operandi” of event files has consequences for the efficacy of response selection and execution. Whenever identical or similar stimuli require different responses, previously established bindings in an event file cause problems because these preestablished stimulus–response bindings and expectancies on stimulus–response associations are only partially fulfilled (Colzato, Warrens, & Hommel, 2006; Hommel, 2004). As a consequence, the event file has to be reconfigured, which slows down responses and increases error rates. This is referred to as partial repetition costs (Colzato, Warrens, & Hommel, 2006; Hommel, 2004). On the contrary, whenever identical or similar stimuli require the same responses, preestablished bindings in an event file facilitate responding. This is referred to as partial repetition benefit (Colzato, Warrens, & Hommel, 2006; Hommel, 2004). However, the neurophysiological mechanisms underlying event file dynamics are unclear. Generally, direct links between neurophysiology and cognitive theoretical frameworks have rarely been established.

Several lines of evidence suggest that event coding or related processes depend on activity in various brain areas encompassing inferior parietal areas, supplementary motor areas, the dorsolateral prefrontal cortex, and the hippocampus (Chmielewski & Beste, 2019a; Chmielewski & Beste, 2019b; Chmielewski & Beste, 2019c; Elsner et al., 2002; Kühn, Keizer, Colzato, Rombouts, & Hommel, 2011; Opitz, Beste, & Stock, 2020; Petruo et al., 2018; Petruo, Stock, Münchau, & Beste, 2016; Zmigrod, Colzato, & Hommel, 2014). It thus seems that event file binding processes are associated with the integration of information across distant brain regions. From a biophysical point of view, and according to the “temporal binding hypothesis” (Crick & Koch, 2003; Varela, 1995; von der Malsburg, 1994), information processing between distant neural assemblies strongly depends on the strength of a coherent organization of activity through synchronous neural oscillations (Buzsáki, 2006; Buzsáki & Draguhn, 2004). More specifically, particularly low‐frequency, high‐amplitude oscillations are suitable to integrate information across spatial distances (Buzsáki & Draguhn, 2004). This is also one reason why theta oscillations have repeatedly been shown to underlie response selection and cognitive control processes (Cavanagh & Frank, 2014; Cohen, 2014). Moreover, theta oscillations were consistently shown in tasks that involve creating and keeping mental representation online, such as the retention of information in working memory, and reorientation or allocation of attention to sensory stimuli (Gevins, Smith, McEvoy, & Yu, 1997; Hsieh, Ekstrom, & Ranganath, 2011; Jensen & Tesche, 2002; Onton, Delorme, & Makeig, 2005; Raghavachari et al., 2001; Tóth et al., 2014). Since event codes depend on keeping information online (Colzato, Raffone, & Hommel, 2006; Hommel, 2009; Hommel et al., 2001), theta oscillations potentially play an important role in event file coding. This also seems to be the case from a network perspective, as a previous study showed that functional connectivity in the theta band promotes the acquisition of frequency‐based stimulus–response associations (Tóth et al., 2017). Furthermore, stimulus–response integration has been implicated in another long‐range frequency band. Namely, alpha frequency oscillations seem to be related to action planning of motor sequences (Bassett et al., 2011; Clarke, Roberts, & Ranganath, 2018; Crivelli‐Decker, Hsieh, Clarke, & Ranganath, 2018; Fell & Axmacher, 2011; Pollok, Latz, Krause, Butz, & Schnitzler, 2014), but not to stimulus‐oriented statistical learning (Tóth et al., 2017). Overall, theta and alpha oscillations have a key role for associative memory formation (Clarke et al., 2018; Crivelli‐Decker et al., 2018), in which theta is predominantly implicated in the formation of stimulus–response associations (Tóth et al., 2017). Therefore, it may be hypothesized that particularly theta band activity reflects binding processes during event coding. Yet, it is important to consider that TEC states that the processing and activation of an event file have to be understood in terms of network dynamics and that an event file, in fact, resembles a network (Hommel, 2011). This network aspect is not well captured by analyzing power of theta frequency oscillations. It thus seems more suitable to analyze theta band activity from a network perspective to understand event file binding processes. From such a network perspective, several lines of evidence suggest that the small‐world metric (Achard & Bullmore, 2007; Bassett & Bullmore, 2006; Bullmore & Sporns, 2009) is a suitable measure to describe EEG network activity (Beste et al., 2019; Vecchio et al., 2018). Small‐world networks are thought to enable the efficient separation *and* functional integration of information (Achard & Bullmore, 2007; Bassett & Bullmore, 2006). Networks with a high level of separation process the information in highly specialized nodes, which are sparsely connected to each other (Achard & Bullmore, 2007; Bassett & Bullmore, 2006). This regular, modular network architecture would be too rigid to dynamically create, retrieve, unbind, and rebind stimulus–response associations. In contrast, networks with a high level of functional integration shift to randomness, as nodes from faraway parts of the network need to be connected to integrate different information (Achard & Bullmore, 2007; Bassett & Bullmore, 2006). An ideal information processing would require a small‐world‐like network, which represents the balance between separation (regularity) and integration (randomness) (Achard & Bullmore, 2007; Bassett & Bullmore, 2006). It is possible, that event file coding relies on a small‐world‐like network; however, further processes of unbinding and reconfiguration would require more integration, and therefore, it may shift the network to randomness. Since these aspects are important for event file processing (Hommel, 2009), the small‐world metric may be of particular relevance in the context of the analysis of neurophysiological processes underlying event file processing. This is all the more the case because it has recently been shown that response selection and control processes affect the organization of theta band activity from the perspective of small‐world networks (Bensmann, Zink, Mückschel, Beste, & Stock, 2019; Beste et al., 2019). We hypothesize that the organization (architecture) of the network is modulated during event file processing. Telesford, Joyce, Hayasaka, Burdette, and Laurienti (2011) proposed a metric in which small‐world values (*ω*) are restricted to the interval −1 to 1 regardless of network size. If *ω* is close to 0, a network organization is considered small‐world and very efficient. Positive *ω* values represent random properties in network organization, negative values indicate that a network has more regular properties in network organization. Both, a more random and a more regular organization reflect a less efficient network organization (Telesford et al., 2011). Previous studies showed that more demanding task conditions shift the network to a more random structure (Beste et al., 2019; Wolff, Zink, Stock, & Beste, 2017). That is, for complex processes, longer path lengths are needed to transmit information across the processing nodes. We hypothesize that the network architecture becomes less efficient when there are partial repetition costs and the network architecture is more small‐world like when there are partial repetition benefits. Taken together, it is likely that particularly network measures of theta band activity reflect event file coding processes.

Another crucial aspect to consider in the context of event file coding is that the EEG signal is composed of different subelements from different sources (Huster, Plis, & Calhoun, 2015; Nunez et al., 1997; Stock, Gohil, Huster, & Beste, 2017). This is particularly the case for event‐related potential (ERP) data. In fact, it has been suggested that ERP correlates of response selection (e.g., N2 and P3) reflect a mixture of different codes related to perceptual processing (“stimulus codes”) and response selection (“response selection codes”) (Chmielewski, Mückschel, Ziemssen, & Beste, 2017; Folstein & Van Petten, 2008; Mückschel, Chmielewski, Ziemssen, & Beste, 2017; Mückschel, Dippel, & Beste, 2017) posing the problem that ERPs may not precisely capture dynamics occurring in event files. From a TEC perspective, though it is important to disentangle binding processes occurring in an event file from stimulus‐ or response‐related processes in the object or action file. Therefore, it may be relevant to isolate (decompose) different components in the ERP signal (Mückschel, Chmielewski, et al., 2017; Mückschel, Dippel, & Beste, 2017; Opitz et al., 2020) to adequately investigate event coding processes. In the TEC context, this decomposition should ideally yield three “clusters” of EEG activity capturing dynamics related to the object, the action file, and the event file. Notably, Ouyang, Herzmann, Zhou, and Sommer (2011); Ouyang, Sommer, and Zhou (2015) proposed a temporal signal decomposition method that decomposes EEG into three clusters of dissociable functional relevance: The S‐cluster refers to stimulus‐related processes (like perception and attention), the R‐cluster refers to response‐related processes (including motor preparation/execution) and the C‐cluster refers to intermediate processes linking S and R (Ouyang et al., 2011; Ouyang et al., 2015; Ouyang, Hildebrandt, Sommer, & Zhou, 2017). Though this temporal decomposition method (i.e., residue iteration decomposition [RIDE]) was originally developed to account for intraindividual variability in EEG data (Ouyang et al., 2011; Ouyang et al., 2015; Ouyang et al., 2017), it has already been shown that it can also be used to dissociate different coding levels in a theoretically meaningful way in EEG data (Mückschel, Chmielewski, et al., 2017; Mückschel, Dippel, & Beste, 2017). Obviously, such clustering reveals striking similarities with the “file structure” proposed by TEC, that is, the S‐cluster may reflect object file related processes, the R‐cluster action file related processes and the C‐cluster event file related processes. Several lines of evidence suggest that particularly the C‐cluster may reflect stimulus–response translation processes (Ouyang et al., 2017; Verleger, Metzner, Ouyang, Śmigasiewicz, & Zhou, 2014; Wolff, Mückschel, & Beste, 2017). Just recently, a study analyzed how distractors and interfering information is processes and how different forms of distracting information affect each other (Opitz et al., 2020). This study was also motivated by the TEC‐framework and reported that distractor bindings also occur in the C‐cluster, as opposed to S‐ and R‐cluster, and undecomposed EEG (Opitz et al., 2020).

Therefore, we hypothesize that event file binding effects are especially reflected by the C‐cluster. In contrast, the S‐cluster and the R‐cluster should not reflect event file binding effects. If this is the case, and because standard ERP‐components reflect a combination of all three clusters (Ouyang et al., 2011; Ouyang et al., 2015; Ouyang et al., 2017), it is reasonable to hypothesize that event file binding effects are stronger when being analyzed at the C‐cluster level, compared to nondecomposed ERPs. Regarding standard ERPs, it is most likely that event file binding processes are reflected by the P3 ERP component. The reason is that especially the P3 ERP‐component has been suggested to reflect processes mediating between stimulus evaluation and responding (Falkenstein, Hohnsbein, & Hoormann, 1994; Mückschel, Stock, & Beste, 2014; Twomey, Murphy, Kelly, & O'Connell, 2015; Verleger, Jaśkowski, & Wascher, 2005), that is, processes central for event file binding according to TEC. Specifically, the P3 reflects the amount of reactivation needed for the established S–R links (Verleger, Hamann, Asanowicz, & Śmigasiewicz, 2015; Verleger, Siller, Ouyang, & Śmigasiewicz, 2017). Moreover, the C‐cluster has been suggested to reflect processes captured by the P3 (Ouyang et al., 2017). Since modulations in the P3 and the C‐cluster have been shown to be associated with activity modulations in inferior parietal areas (Mückschel et al., 2014; Verleger, Heide, Butt, & Kömpf, 1994; Wolff, Mückschel, & Beste, 2017; Wolff, Mückschel, Ziemssen, & Beste, 2018), we hypothesize that activity in these regions is also modulated during event file binding.

## 2 METHODS

### 2.1 Participants

A sample of *N* = 28 (9 males and 18 females, *M*_age_ = 23.4, *SD*_age_ = 3.3 years) healthy young adults participated in the study. Comparable sample sizes have been used in other EEG studies using the TEC framework (i.e., *N* = 27 in Opitz et al., 2020) and which also used nearly identical experimental paradigms (*N* = 23 in each group of a between‐subject design on event file coding reported by Petruo et al., 2016). All participants had normal or corrected‐to‐normal vision. They did not report any history of psychiatric or neurological disorders or taking centrally acting medication. All participants were undergraduate or graduate students and received financial reimbursement for their participation. All participants gave written informed consent prior to their study participation. The study was carried out in accordance with the declaration of Helsinki. The study was approved by the ethics committee of the TU Dresden. We performed a sensitivity analysis in G × Power (Faul, Erdfelder, Lang, & Buchner, 2007), in which a sample size of *N* = 28 with a power of .95 resulted a required effect size of *f* = .28. As *f* = .28 is equivalent to *η*_p_^2^ = .08, we consider effects reliable if they are larger than *η*_p_^2^ = .08. As can be seen in the Section 3, significant effects at the behavioral and neurophysiological level are stronger than this threshold and, therefore, reflect reliable effects.

### 2.2 Task

**Figure 1:**
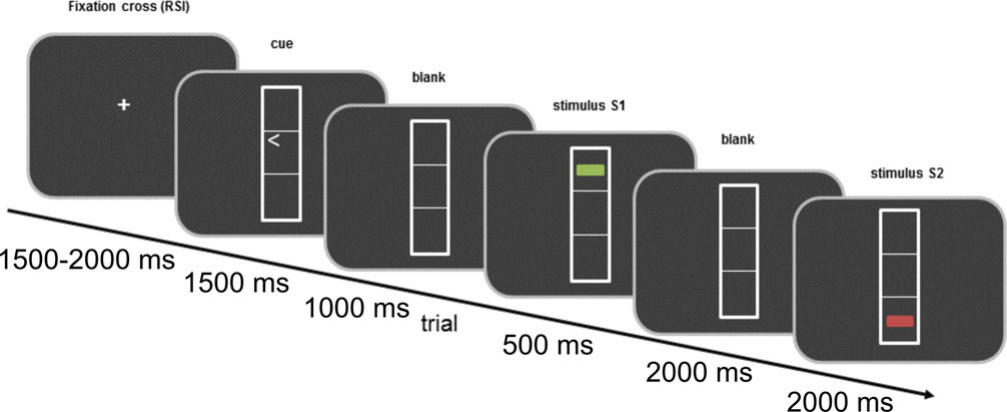
Schematic illustration of the paradigm. The figure represents the order of the stimuli during the trial. The timing of the stimuli is described in the text

Event file coding was examined by using an event file coding paradigm (Hommel, 1998) or also known as an S–R task (Colzato, Warrens, & Hommel, 2006). The event file or task is depicted in Figure 1.

Participants were seated in front of a 17‐in. CRT screen, at a distance of 60 cm. During the trials, participants saw three vertically aligned boxes in the middle of a screen. Each box had a size of 2.4 × 0.9 cm^2^. In the middle box, participants saw a left‐ or right‐pointing arrowhead, which represented the response cue. It was followed by a vertical and a horizontal line presented in vertically aligned boxes similar to the response cues. The lines could be red or green and could be placed in the top box or in the bottom one. These lines served as Stimulus 1 (S1) and Stimulus 2 (S2). Importantly, S1 varied randomly in orientation (vertical or horizontal), location (top or bottom), and color (red or green). Similarly, S2 consisted of the same randomly varied features as S1. Thus, in some trials, none of these features were shared between S1 and S2 (no feature overlap condition), other trials presented identical S1 and S2 (full feature overlap condition), and the remaining trials shared one or two features from the available three between the stimuli (partial feature overlap conditions: one feature and two feature overlap). Two responses (R1 and R2) had to be executed per trial by pressing the left or right control key of a computer keyboard with the corresponding index finger. Thus, two consecutive responses could require the same button press (response repetition), or two different ones (response alternation). Participants were informed that there would be no systematic relationship between S1 and R1, or between S1 and S2. Thus, the task was designed to study automatic binding effects, that is, interactions between repetitions of stimulus features (stimulus feature overlap) and responses. The timing of the experiment was the following: in every trial, first the cue appeared on the screen for 1,500 ms. Participants were instructed not to react immediately to the cue, but rather withhold their response until the presentation of S1. After the response cue, a blank screen was displayed for 1,000 ms. It was followed by S1 shown for 500 ms. After the appearance of S1, participants were expected to carry out R1 (right keypress when the cue was pointing to the right and vice versa). Importantly, R1 was carried out simultaneously to but independently of the orientation, color, or location of S1. This notwithstanding, the close proximity of S1 and R1 causes S1 to become related to R1 (automatic binding). A blank screen for 2,000 ms followed the presentation of S1. Next, S2 was presented for 2,000 ms or until a response was given. R2 required a response to the shape of S2 (vertical vs. horizontal). Participants were instructed to press the left key when a horizontal and the right key when a vertical line was shown. If the R1 was incorrect, the trial was repeated once. The whole session comprised 384 trials, which exceeded to the maximum of 395 due to the repetition of erroneous R1s. The number of trials was determined as a factorial combination of S2 features, such as shape (2) × color (2) × location (2), the repetition versus alternation of shape (2) × the repetition versus alternation of color (2) × the repetition versus alternation of location (2) × and response (2), × each combination repeated three times (Colzato, Warrens, & Hommel, 2006). During intertrial intervals, which were jittered between 1,500 and 2,000 ms a fixation cross was presented in the middle of the screen. Importantly, unlike in the “voluntary” version of the task (Colzato, Warrens, & Hommel, 2006; Petruo et al., 2016), participants were not tested on their knowledge of the S1. Therefore, “automatic event file binding” was tested (Hommel, 1998; Hommel, 2005).

### 2.3 EEG recording analysis

The EEG was recorded from 60 Ag/AgCl electrodes (EasyCap, Germany) in equidistant positions using a QuickAmp amplifier and the Brain Vision Recorder 1.2 software (Brain Products, Germany). The remaining EOG channels were disabled for the recording. The ground and reference (Fpz) electrodes were placed at coordinates *θ* = 58, *φ* = 78 and *θ* = 90, *φ* = 90, respectively. The sampling rate was 500 Hz. All electrode impedances were kept below 5 kΩ. Data preprocessing was performed by using Brain Vision Analyzer (Brain Products) and involved the following steps: First, the data were downsampled to 256 Hz and band‐pass filtered (IIR filter: 0.5–20 Hz, an order of 8). The downsampled data were rereferenced to an average reference. Then, a manual inspection of the data was carried out to remove technical artifacts. Remaining artifacts with periodical effects such as blinks, eye movements, and pulse artifacts were removed by an independent component analysis (Infomax algorithm). Components (7.57 *±* 2.27) have been removed if they showed identifiable spectrum and topography, such as vertical and horizontal eye movements, cardiovascular artifacts, or muscular activity. The preprocessed data were segmented by using epochs locked on the S2 (−1,000 to 1,000 ms). While event file binding originally occurs after the establishment of the S1‐R1 link, the binding has been traditionally studied in terms of the retrieval, unbinding, and reconfiguration, which is necessitated by the S2‐R2 (Hommel, 1998; Hommel, 2004; Kühn et al., 2011). Only trials with correct R1 and R2 responses were included in the segmentation. Separate segments were created for all combinations of feature overlap levels (no, one feature overlap, two features overlap, and full overlap between S1 and S2 stimulus features) and responses (repetition vs. switch).

On the segmented data, an automated artifact rejection procedure was applied in the time window of 1,000 ms before and after the S2. This process discarded all segments with amplitudes higher than 150 μV, or lower than −150 μV, or activities lower than 0.5 μV over a time interval of at least 100 ms. To obtain reference‐free neurophysiological data, a current source density (CSD) transformation was applied (Kayser & Tenke, 2015; Perrin, Pernier, Bertrand, & Echallier, 1989). This transformation uses the potential difference between one electrode and the total potential of all surrounding electrodes. Thus, the CSD transformation also serves as a spatial filter highlighting scalp topography, which helps to identify electrodes that best reflect activity related to experimental conditions (Nunez, Pilgreen, Westdorp, Law, & Nelson, 1991; Tenke & Kayser, 2012). A baseline correction was applied to a time interval of −200 to 0 ms prior to the S2 stimulus onset. Next, averages were computed separately for each condition and participant. Based on the a priori hypotheses, especially the P3 ERP component is of interest. To analyze the stimulus‐locked P3, we selected the electrode Cz, based on the scalp topography. After the electrode selection, we determined the time window of the P3 component by visual inspection: 400–700 ms after the S2 presentation. Within this time interval, the mean amplitude was quantified and extracted at the single‐subject level. This choice of electrodes and time window was validated using the statistical method proposed by Mückschel et al., 2014: the amplitude in above‐mentioned time window was extracted for all 60 electrodes. Each electrode was subsequently compared to the average of all other electrodes using Bonferroni correction for multiple comparisons (critical threshold *p* = .0008). Only electrodes that showed significantly larger mean amplitudes (i.e., negative for N‐potentials and positive for the P‐potentials) than the remaining electrodes were selected.” This procedure confirmed the choice of electrode Cz.

### 2.4 Residue iteration decomposition

RIDE postulates that different components with variable intercomponent delays can be differentiated within ERPs (Ouyang et al., 2015). Based on this assumption, RIDE decomposes single‐trial ERPs into different components with static or variable latencies. According to the timing and variability of these components, they can be linked to different stages of information processing. RIDE uses an iterative temporal decomposition, which has been used with robust results before (Mückschel, Chmielewski, et al., 2017; Ouyang et al., 2015). Decomposition is employed for each electrode separately; therefore, it is sensitive to the channel‐specific latency variability information (Ouyang et al., 2015). Since RIDE performs the decomposition irrespective of the scalp distributions (Ouyang et al., 2015), the CSD reference does not influence the results. In the current study, RIDE decomposition was performed according to established procedures (Chmielewski et al., 2017; Ouyang et al., 2011; Verleger et al., 2014) using the RIDE toolbox (for a manual, see http://cns.hkbu.edu.hk/RIDE.htm) in MATLAB (MathWorks, Inc., Natick, MA). We used latency information relative to the stimulus and response onsets to derive the S (“stimulus”) and R (“response”) clusters. The C (“central”) cluster's latency information is estimated in every single‐trial and iteratively improved. RIDE requires predefined time windows to extract the waveforms for each cluster (Ouyang et al., 2015; Ouyang, Schacht, Zhou, & Sommer, 2013). RIDE requires predefined time windows to extract the waveform of each RIDE component. Each of these time windows should cover the range within each component is expected to occur (Ouyang et al., 2013; Ouyang et al., 2015). That is, the R‐cluster should occur around the response; the S‐cluster should cover the stimulus presentation and the subsequent processes from P1 to N2; and finally, the C‐cluster should cover the time windows of P2, N2, and P3. We applied the following intervals: for the S‐cluster, 200 ms prior to S2 and to 700 ms after the S2 presentation; for the R‐cluster, 300 ms before and after the R2; for the C‐cluster, 150–800 ms after the S2 stimulus. Using the provided markers, RIDE uses an iterative decomposition with an L1‐norm minimization, which creates median waveforms. For the estimation of the S‐cluster, RIDE subtracts C and R from each trial and aligns the residual of all trials to the latency information of S. The result is the median waveform for all time points in the S‐cluster interval. The same procedure is followed to derive clusters C and R. The whole process is iterated to improve the estimation of the components until they converge. Further details of the RIDE method can be found in Ouyang et al. (2015, 2011). After obtaining the RIDE clusters, we used the same mean amplitude extraction method as described above for the standard ERPs. As the current study's focus is on the P3 component, we present the analyses related to the C‐cluster, where P3 was the most visible (see Figure 3). The validation procedure, as also performed for the ERP data, confirmed the site of the chosen electrode and time window.

### 2.5 Source localization

Source localization was used to examine the source of the interaction “feature overlap × response” for the ERP data and the RIDE‐decomposed data. For that, the standard low‐resolution brain electromagnetic tomography (sLORETA) algorithm was used (Pascual‐Marqui, 2002). It requires standard electrode coordinates according to the 10/10 or 10/20 system as input. The method uses a three‐shell spherical head model and the covariance matrix was calculated using the single subject's baseline. Within this head model, the intracerebral volume is partitioned into 6,239 voxels using a spatial resolution of 5 mm and the standardized current density is calculated for every voxel, using an MNI152 head model template. The algorithm provides a single linear solution for the inverse problem without localization bias (Marco‐Pallarés, Grau, & Ruffini, 2005; Pascual‐Marqui, 2002; Sekihara, Sahani, & Nagarajan, 2005). The validity of sLORETA results has been shown in combined fMRI/EEG and TMS/EEG studies (Dippel & Beste, 2015; Sekihara et al., 2005). For the sLORETA contrasts, we performed a comparison against zero. To calculate the statistics on the sLORETA sources (contrasts), we utilized voxel‐wise randomization tests with 2,500 permutations and statistical nonparametric mapping procedures (SnPM). Locations of voxels that were significantly different (*p* < .05) are shown in the MNI‐brain www.unizh.ch/keyinst/NewLORETA/sLORETA/sLORETA.htm. Activations shown in the brain represent critical *t* values corrected for multiple comparisons.

### 2.6 Small‐world network analysis

The analysis of the small‐world network architecture was performed similarly to previous studies (Bensmann et al., 2019; Beste et al., 2019; Wolff, Zink, et al., 2017). In the preprocessed, segmented data, the entire spectral content (i.e., the power spectrum) in a frequency range of interests (i.e., theta at 4–8 Hz and alpha at 8–12 Hz) was analyzed. The imaginary part of the coherence spectrum was calculated for all possible EEG electrode pairs in the theta and alpha frequency bands (Nolte et al., 2004). Then, the binary adjacency network matrices (based on all electrodes) were calculated. If the coherence between two electrodes was “strong,” an unweighted and undirected connection was defined represented by 1. If the coherence between two electrodes was “weak,” this was represented by 0. To define which coherence is “strong” or “weak,” only the highest 15 or 10% strongest connections were included in the analysis, referred to as 85 and 90% threshold conditions, respectively. This approach has been used previously in the context of response selection and inhibitory control (Bensmann et al., 2019; Beste et al., 2019) and is a compromise to cope with two problems: On the one hand, it ensures that only electrodes with high coherence are defined as being “connected” and included in the analysis. On the other hand, it also ensures that enough connections are left to form an electrode network (Wolff, Zink, et al., 2017; Zink, Stock, Colzato, & Beste, 2018). While there are several ways to determine the threshold, for instance, based on some statistical parameterization and previous observation in the literature, all of them remain arbitrary (Langer, Pedroni, & Jäncke, 2013). Then, a binary 60 × 60 adjacency network matrix (based on the 60 EEG electrodes) was calculated. In this matrix, 1 represents an unweighted and undirected connection between any pair of electrodes and 0 represents no connection. We used the Watts and Strogatz method to study small‐world networks (Watts & Strogatz, 1998). As done in previous studies, this method was applied to each single subject (Beste et al., 2019; Wolff, Zink, et al., 2017; Zink et al., 2018): Using this method, one starts from a one‐dimensional network, where each node in the network (in the current study, the EEG electrode) is only connected to its *k* nearest neighbors on either side, representing a “regular” network with randomness 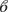 = 0, a ring lattice with *N* nodes of mean degree 2*k* is created. Next, more connections (“edges”) are randomly chosen to another random node with increasing randomness (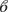 *>* 0*)*. When 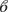 = 0, no edges are rewired and the model returns a ring lattice. In contrast, when 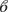 = 1, all of the edges are rewired and the ring lattice is transformed into a random network containing *N* nodes and mean node degree of 2*k*. A network has small‐world network properties when it has properties of lattice networks showing clustered interconnectivity (i.e., high clustering coefficient, “C”) and properties of random networks showing short geodetic distance (i.e., a short average path length, “L”). Regular networks have a high C and a high L. Random networks have a low C and a low L. Therefore, neither regular nor random networks alone can explain a small‐world network architecture (Watts & Strogatz, 1998). For every single subject, the average number of edges from one node to all other nodes (degree, *2k*), average shortest path length (geodetic distance, *L*_real_) and average clustering coefficient (*C*_real_) were calculated. For each individual, completely random (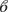 = 0) and completely regular (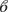 = 1), Watts–Strogatz models were created and *L*_rand_ and *C*_rand_ and *C*_latt_ were also computed. We analyzed all small‐world values (*ω*) according to Telesford et al. (2011), who proposed a quantitative categorical definition of a small‐world network in line with the definitions of the original Watts–Strogatz model. The parameter *ω* is calculated by:

$$\omega =\frac{L_{\mathrm{rand}}}{L}-\frac{C}{C_{\text{latt}}}$$

In the formula, “rand” refers to a random network and “latt” to a lattice network, which is constructed on the basis of the measured data. Small‐world values of *ω* are restricted to the interval −1 to 1 regardless of network size. If *ω* is close to 0, it is considered as small world. Positive *ω* values represent more random properties, negative values indicate that a network has more regular or lattice‐like properties. Both, a more random and a more regular organization reflect a less efficient network organization (Telesford et al., 2011).

### 2.7 Statistics

Statistical analyses were performed by using JASP 0.11.1 (Love et al., 2019). Mean accuracy (percentage of correct responses) and medians of RT data (for correct responses) were calculated for each participant and each condition. To examine event file coding, accuracy and RT data were analyzed in two‐way repeated measures analysis of variance (ANOVA) with feature overlap (no, one feature overlap, two features overlap, and full overlap between S1 and S2 stimulus features) and response (repetition vs. switch) as within‐subject factors. This approach is identical to previous studies examining binding effects in the event coding framework (Beste et al., 2016; Petruo et al., 2016). The average number of trials (together with the minimum and maximum numbers, respectively) considered for statistical analyses were the following: no feature overlap repetition *M* = 22, (14–26); no feature overlap alternation *M* = 23, (12–26); one feature overlap repetition *M* = 68, (44–74); one feature overlap alternation *M* = 69, (48–72); two features overlap repetition *M* = 68, (45–72); two features overlap alternation *M* = 68, (47–72); full overlap repetition *M* = 23, (19–24); full overlap alternation *M* = 23, (15–29). After inspecting the behavioral results (see Section 3.1), it was revealed that the difference between the no feature overlap and the full feature overlap conditions shows the strongest binding effect at the level of accuracy and the reaction times. As the goal of the study was to provide in‐depth analyses of event file coding and binding at the neurophysiological level, and we did not formulate hypotheses on the partial overlap conditions, we included no feature overlap and full feature overlap conditions, but not the partial feature overlap conditions in the neurophysiological analyses. Thus, the mean amplitude and small‐world value data were analyzed in two‐way repeated measures ANOVA with feature overlap (full vs. no feature overlap) and response (repetition vs. switch) as within‐subject factors. The average number of trials (together with the minimum and maximum numbers, respectively) considered for statistical analysis of the ERPs was the following: no feature overlap repetition *M* = 15.4, (7–22); no feature overlap alternation *M* = 18, (10–23); full overlap repetition *M* = 17, (11–23); full overlap alternation *M* = 14, (7–23). While these trial numbers might be considered low for conventional ERP analysis, they are in line with previous ERP results of event file coding (Petruo et al., 2016). Most important, since RIDE uses L1‐norm‐based method and has implemented several routines to decrease across‐trial intraindividual variability in the data (see above), it leads to higher consistency of neurophysiological processes within subjects (i.e., across trials) than the more common L2‐norm‐based procedures, which is the classical ERP averaging approach (Ouyang et al., 2013; Ouyang et al., 2015). Therefore, lower trial numbers allow to obtain reliable effects. Finally, a limited amount of practice in the task is required to avoid the confound of possible learning effects in event file coding (Colzato, Raffone, & Hommel, 2006; Eberhardt, Esser, & Haider, 2017; Hommel & Colzato, 2009). Here, we report *η* ^2^ effect size for ANOVA main effects and interactions, and confidence interval of 90% for the effect sizes (Steiger, 2004). Moreover, the Bayes factor as BF_10_ is reported to quantify the evidence for the alternative hypothesis. The default JASP prior for fixed effects was used (*r* scale prior width = 0.5). All post hoc tests were Bonferroni corrected.

## 3 RESULTS

### 3.1 Behavioral data

**Figure 2:**
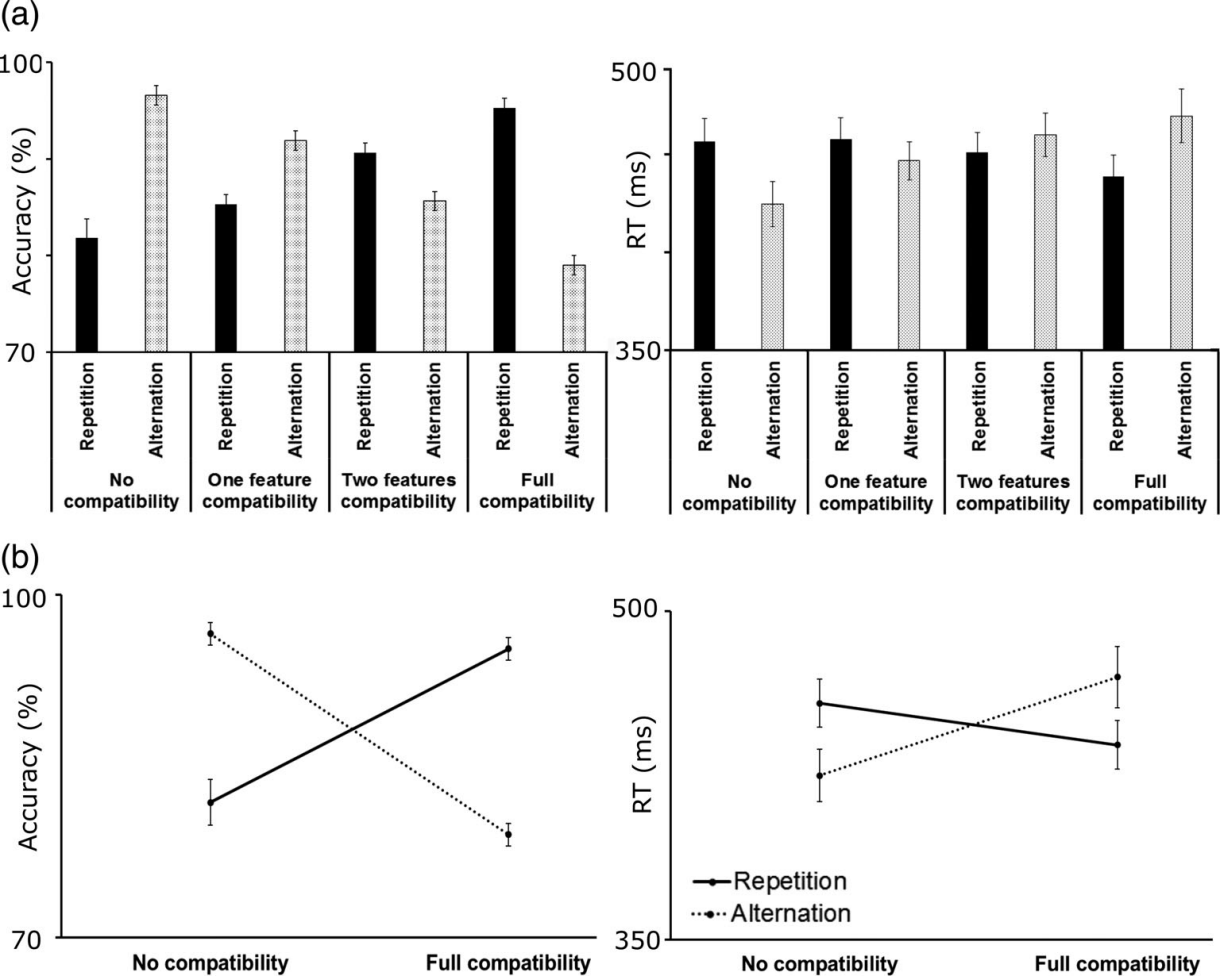
Behavioral results across feature overlap and response type. (a) Mean accuracy (left) is shown as a function of feature overlap (i.e., number of overlapping features) for repeated and alternated responses. Mean RT (right) is shown as a function of feature overlap for repeated and alternated responses. Repeated responses are indicated by black bars, alternated responses are indicated by dotted bars. (b) Binding (interaction) effects for mean accuracy and for mean RT in the no feature overlap and full feature overlap condition. Repeated responses are indicated by black lines, alternated responses are indicated by dotted lines. Error bars denote *SE* of mean

The behavioral data are shown in Figure 2.

The feature overlap by response ANOVA on the accuracy data showed that the main effect of feature overlap was significant (*F*(3,81) = 3.16, *p* = .029, *η*_p_^2^ = .105, 90% CI [.006; .192], BF_10_ = 1.75). Participants were more accurate in the no feature overlap than the full feature overlap condition (89.21% *±* 1.09 vs. 86.16% *±* 1.54, *p* = .040); furthermore, to a lesser degree, participants were more accurate in the one feature overlap (88.61% *±* 1.32, *p* = .037) and the two features overlap conditions (88.14% *±* 1.50, *p* = .039) than in the full feature overlap condition. All other pairwise comparisons were not significant (*p* > .05). In contrast, the main effect of response was not significant (*F*(1,27) = 0.16, *p* = .693, *η*_p_^2^ = .006, 90% CI [.000; .117], BF_10_ = 0.16). Importantly, the feature overlap by response interaction was significant (*F*(3,81) = 56.25, *p* < .001, *η*_p_^2^ = .676, 90% CI [.566; .732], BF_10_ = 8.21). When responses had to be repeated, accuracy increased from the no feature overlap (81.82% *±* 1.96) to the two features overlap (90.64% *±* 1.34, *p* < .001) and the full feature overlap (95.29% *±* 1.14, *p* < .001) conditions. In contrast, accuracy decreased from the no feature overlap (96.61% *±* .83) to the one feature overlap (91.89% *±* 1.16, *p* = .004), to the two features overlap (85.64% *±* 1.96, *p* < .001), and the full feature overlap conditions (78.64% *±* 1.34, *p* < .001) when response had to be alternated. Additionally, with response alternation, the full feature overlap condition showed lower accuracy than the one feature (*p* < .001) and two features overlap conditions (*p* < .001), respectively. All other pairwise comparisons were not significant (*p* > .05).

The feature overlap by response ANOVA on the RT data showed that the main effect of feature overlap was significant (*F*(3,81) = 4.61, *p* = .005, *η*_p_^2^ = .146, 90% CI [.028; .241], BF_10_ = 4.47). Participants were faster at the null feature overlap (441 ms *±* 11) than at one feature overlap (452 ms *±* 10, *p* = .021) or at the two features overlap conditions (456 ms *±* 10, *p* = .003). In contrast, the main effect of response was not significant (*F*(1,27) = 0.07, *p* = .795, *η*_p_^2^ = .003, 90% CI [.000; .091], BF_10_ = 0.15). The feature overlap by response interaction was significant (*F*(3,81) = 29.15, *p* < .001, *η*_p_^2^ = .519, 90% CI [.376; .599], BF_10_ = 2.91). The average RT decreased from the one feature overlap to the full feature overlap condition when response had to be repeated (458 ms *±* 11 vs. 439 ms *±* 11, *p* = .006). In contrast, responses became slower from the no feature overlap (425 ms *±* 12) to the one feature overlap (447 ms *±* 10, *p* = .001), the two features overlap (460 ms *±* 11, *p* < .001), and the full feature overlap conditions (470 ms *±* 14, *p* < .001) when response had to be alternated.

In sum, the behavioral data showed a robust interaction between feature overlap and response type. Specifically, the accuracy analysis revealed a binding effect. A larger feature overlap between S1 and S2 facilitated a higher accuracy if the response had to be repeated, and a lower accuracy if the response had to be alternated. The latter represents *partial repetition costs*. As the differences were largest for the contrasts between no feature overlap and full feature overlap in response repetition conditions and in response alternation conditions, these two levels of the feature overlap condition can be used as reliable indices for the binding effect.

### 3.2 Neurophysiology

To examine neurophysiological correlates of the binding effect observed at the behavioral level, we analyzed the conditions no feature overlap and full feature overlap. This was done because these conditions maximize the assessable binding effect and maximize power/reliability in the neurophysiological data analysis.

#### 3.2.1 Time domain analyses

**Figure 3:**
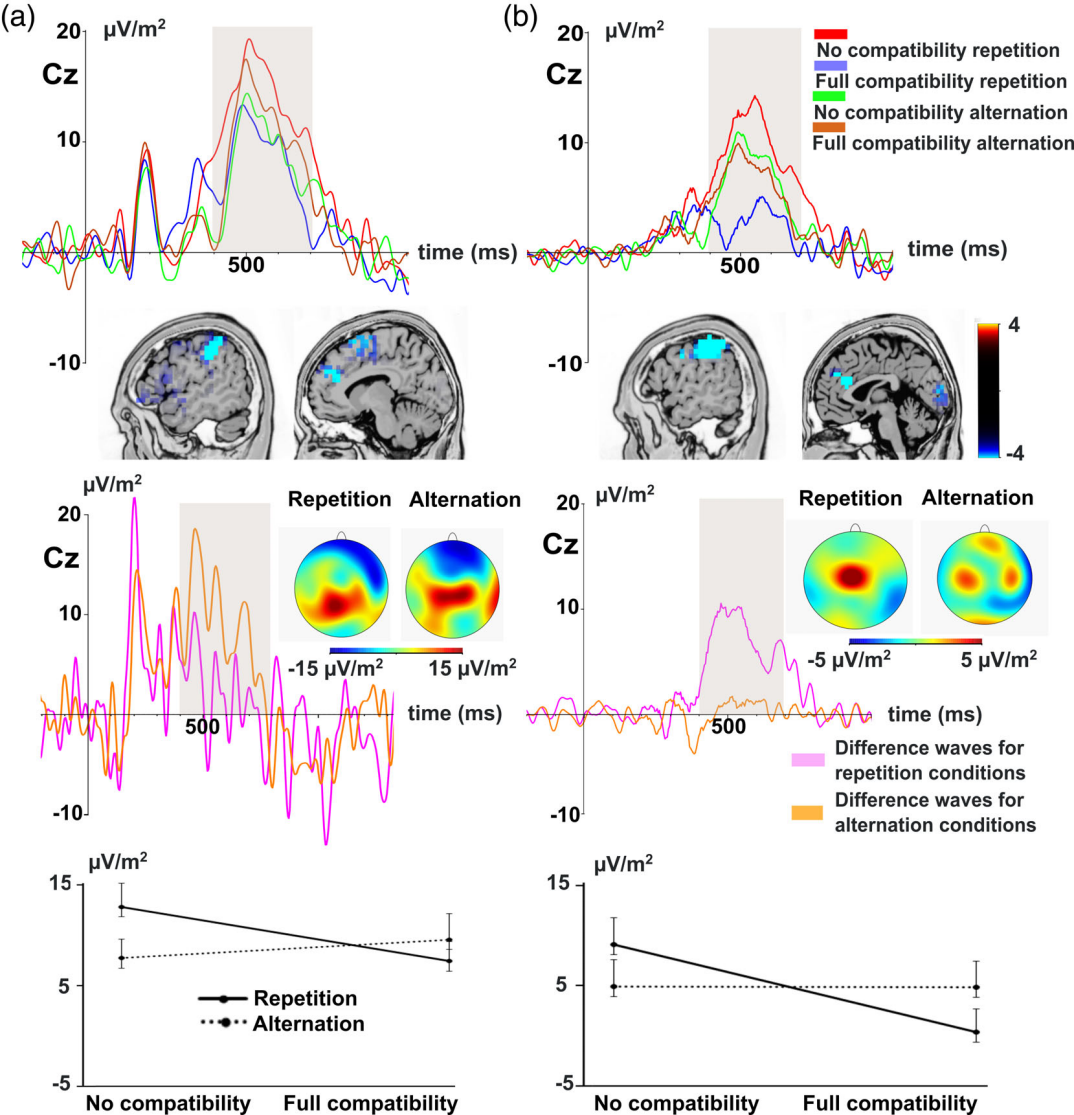
Time‐domain level results. Time point 0 denotes the stimulus presentation. The analyzed time window is marked with a gray shaded area. (a) The standard event‐related potential (ERP) results. The standard P3 ERP component is shown across four conditions: no feature overlap repetition (red), full feature overlap repetition (blue), no feature overlap alternation (green), and full feature overlap alternation (brown). Voxels with significant differences for the binding effects according to the standard low resolution brain electromagnetic tomography (sLORETA) analysis are presented. The sLORETA color bar shows critical *t* values. Difference waves are depicted for response repetition at no feature overlap and between full feature overlap (pink), and response alternation at no feature overlap and between full feature overlap (orange). The scalp topography plots show the distribution of the mean activity of the respective difference wave areas. The line graph shows the interaction between the binding conditions for the standard ERP data. (b) The decomposed C‐cluster results. The C‐cluster P3 is shown across the four experimental conditions, followed by the significant voxel activations in the sLORETA analysis. Difference waves for response repetition at no feature overlap and between full feature overlap, and response alternation at no feature overlap and between full feature overlap are presented for the C‐cluster. The scalp topography plots show the distribution of the mean activity of the respective difference wave areas for the C‐cluster. The line chart depicts the interaction between the binding conditions for the C‐cluster data

Grand‐average ERP waveforms in the P3 time window split by feature overlap and response type are presented in Figure 3.

The feature overlap by response ANOVA on the mean amplitude of P3 showed that the main effect of feature overlap (*F*(1,27) = 2.76, *p* = .108, *η*_p_^2^ = .093, 90% CI [.000; .277], BF_10_ = 0.46), and the main effect of response were not significant (*F*(1,27) = 3.40, *p* = .076, *η*_p_^2^ = .112, 90% CI [.000; .230], BF_10_ = 0.35). However, the feature overlap by response interaction was significant (*F*(1,27) = 4.63, *p* = .041, *η*_p_^2^ = .146, 90% CI [.035; .338], BF_10_ = 8.74). The mean amplitude of the P3 decreased from the no feature overlap to the full feature overlap condition when response had to be repeated (12.82 μV/m^2^ *±* 12.47 vs. 7.45 μV/m^2^ *±* 10.14, *p* = .007, *d* = .551) but not when the response had to be alternated (7.75 μV/m^2^ *±* 9.94 vs. 9.59 μV/m^2^ *±* 13.39, *p* = .393, *d* = −.164). The sLORETA analysis (see Figure 3) revealed that this interaction effect was reflected by activation modulations in the left inferior parietal cortex (BA40; MNI [x,y,z]: −52, −38, 43), the superior frontal gyrus (BA6; MNI [x,y,z]: −18, 11, 67) and the medial frontal gyrus (BA9; MNI [x,y,z]: −9, 38, 23). The given coordinates reflect the locations of maximal activity (*p* < .05; corrected for multiple comparisons in SnPM).

Grand‐average ERP waveforms for the three RIDE clusters split by feature overlap and response type are presented in Figure 3. The feature overlap by response ANOVA for the mean amplitude in the C‐cluster P3 time window showed that the main effect of feature overlap was significant (*F*(1,27) = 7.45, *p* = .001, *η*_p_^2^ = .216, 90% CI [.301; .407], BF_10_ = 2.45). The amplitude was larger for the no feature overlap (7.02 μV/m^2^ *±* 14.03) than for the full feature overlap condition (2.61 μV/m^2^ *±* 12.92). In contrast, the main effect of response was not significant (*F*(1,27) = 0.011, *p* = .918, *η*_p_^2^ = .001, 90% CI [.000; .016], BF_10_ = 0.20). Finally, the interaction between feature overlap and response type was significant (*F*(1,27) = 7.12, *p* = .013, *η*_p_^2^ = .209, 90% CI [.027; .400], BF_10_ = 10.31). The mean amplitude of the C‐cluster P3 decreased from the no feature overlap to the full feature overlap condition when response had to be repeated (9.08 μV/m^2^ *±* 14.08 vs. 0.38 μV/m^2^ *±* 12.12, *p* < .001, *d* = .744) but not when the response had to be alternated (4.95 μV/m^2^ *±* 13.98 vs. 4.84 μV/m^2^ *±* 13.71, *p* = .961, *d* = −.009). The sLORETA analysis (see Figure 3) revealed that this interaction effect was reflected by activation modulations in the left inferior parietal cortex (BA40; MNI [x,y,z]: −48, −34, 36) and the anterior cingulate cortex (BA24; MNI [x,y,z]: −2, 29, 23).

Regarding the R cluster in the P3 time window (refer Supplemental Figure S1), the feature overlap by response ANOVA on the mean amplitude of P3 showed that the main effects of feature overlap (*F*(1,27) = .098, *p* = .757, *η*_p_^2^ = .004, 90% CI [.000; .101], BF_10_ = 0.14) and response (*F*(1,27) = .031, *p* = .861, *η*_p_^2^ = .001, 90% CI [.000; .044], BF_10_ = 0.14) were not significant. Similarly, the interaction between feature overlap and response type was not significant (*F*(1,27) = .076, *p* = .784, *η*_p_^2^ = .003, 90% CI [.000; .094], BF_10_ = 0.01). The Bayes factor supports the H_0_ over the H_1._ Furthermore, regarding the S‐cluster in the P3 time window (refer Supplemental Figure S1), the feature overlap by response ANOVA on the mean amplitude showed that the main effects of feature overlap (*F*(1,27) = 1.132, *p* = .297, *η*_p_^2^ = .040, 90% CI [.000; .203], BF_10_ = 0.24) and response (*F*(1,27) = .695, *p* = .412, *η*_p_^2^ = .025, 90% CI [.000; .175], BF_10_ = 0.19) were not significant. Similarly, the interaction between feature overlap and response type was not significant (*F*(1,27) = .161, *p* = .692, *η*_p_^2^ = .006, 90% CI [.000; .117], BF_10_ = 0.03). The Bayes factor for the feature overlap by response interaction in the S‐cluster supports the H_0_ over the H_1_.

For information about the P1 and N1 ERP components reflecting perceptual and attentional processes (Herrmann & Knight, 2001) in the S‐cluster, please refer to the supplemental material (Supplemental Figure S2). In short, none of these ERP components revealed interactions indicating binding effects. The same was the case when examining RIDE clusters in these time windows.

#### 3.2.2 Network analyses

As explained in Section 2, TF‐decomposition and network analyses can only reliably be calculated for the nondecomposed EEG data. For the TF‐decomposition method and results, please, see the Supplemental Material.

**Figure 4:**
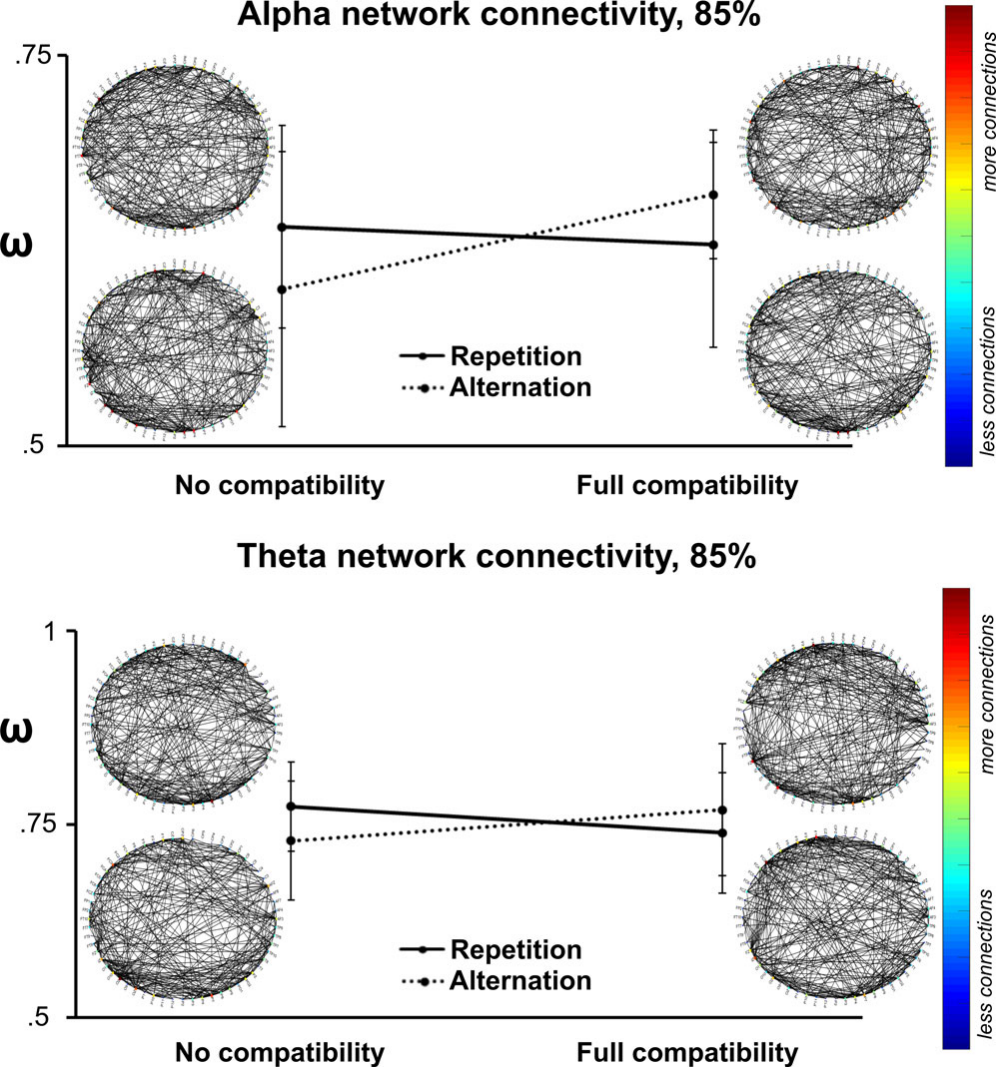
Connectivity results. Alpha and theta oscillation‐based networks are illustrated for the four experimental conditions. The graphs represent the threshold of 85%. The imaginary part of the coherence is plotted as edges between the electrodes (nodes). The clockwise order of the nodes are: CPz, CP6, CP5, CP4, CP3, CP2, CP1, C6, C5, C4, C3, AFz, AF8, AF7, AF4, AF3, TP9, TP8, TP10, T8, T7, Pz, PO2, PO1, P9, P8, P7, P4, P3, P2, P12, P11, P10, P1, Oz, O9, O2, O10, O1, Iz, Fz, FT9, FT8, FT7, FT10, FP2, FP1, FCz, FC6, FC5, FC4, FC3, FC2, FC1, F6, F5, F2, F1, Cz. The color bar denotes the number of connections from one electrode to other nodes. The line chart represents the interaction between the binding conditions for the small‐world values (*ω*)

Small‐world network analyses are shown in Figure 4.

For the 90% threshold in the theta band, the network analysis showed that the main effects of feature overlap (*F*(1,27) = .063, *p* = .804, *η*_p_^2^ = .002, 90% CI [.000; .085], BF_10_ = 0.21) and response (*F*(1,27) = .742, *p* = .397, *η*_p_^2^ = .027, 90% CI [.000; .178], BF_10_ = 0.20) were not significant. However, the significant feature overlap by response interaction was significant (*F*(1,27) = 8.69, *p* = .007, *η*_p_^2^ = .244, 90% CI [.044; .432], BF_10_ = 4.21). Further post hoc *t* tests showed that when response had to be repeated, *ω* were larger in the no feature overlap condition (*ω* = 0.633 *±* 0.046) as compared to the full feature overlap condition (*ω* = 0.601 *±* 0.071) (*t*(27) = 2.21; *p* = .018). When response had to be switched, the parameter *ω* was smaller in the no feature overlap condition (*ω* = 0.607 *±* 0.073) as compared to the full feature overlap condition (*ω* = 0.645 *±* 0.068) (t(27) = 1.97; *p* = .029). For the 85% threshold in the theta band, the network analysis showed a similar pattern. The main effects of feature overlap (*F*(1,27) = .055, *p* = .816, *η*_p_^2^ = .002, 90% CI [.000; .075], BF_10_ = 0.20) and response (*F*(1,27) = .284, *p* = .598, *η*_p_^2^ = .010, 90% CI [.000; .136], BF_10_ = 0.23) were not significant. However, the feature overlap by response w interaction (*F*(1,27) = 4.88, *p* = .036, *η*_p_^2^ = .153, 90% CI [.006; .345], BF_10_ = 7.04) was significant. Further post hoc *t* tests showed that when response had to be repeated, *ω* were larger in the no feature overlap condition (*ω* = 0.773 *±* 0.058) as compared to the full feature overlap condition (*ω* = 0.739 *±* 0.078) (*t*(27) = 1.83; *p* = .039). Again, when response had to be switched, parameter *ω* was smaller in the no feature overlap condition (*ω* = 0.729 *±* 0.077) as compared to the full feature overlap condition (*ω* = 0.769 *±* 0.085) (*t*(27) = 1.98; *p* = .029).

For the alpha frequency band, and using the 90% threshold, the network analysis showed that the main effects of feature overlap (*F*(1,27) = 1.204, *p* = .282, *η*_p_^2^ = .043, 90% CI [.000; .207], BF_10_ = 0.38) and response (*F*(1,27) = .023, *p* = .880, *η*_p_^2^ = .001, 90% CI [.000; .033], BF_10_ = 0.21) were not significant. Similarly, the response by feature overlap interaction was not significant (*F*(1,27) = .849, *p* = .365, *η*_p_^2^ = .030, 90% CI [.000; .185], BF_10_ = .37). For the 85% threshold in the alpha band, the network analysis showed that the main effect of feature overlap (*F*(1,27) = 5.086, *p* = .032, *η*_p_^2^ = .159, 90% CI [.014; .370], BF_10_ = 7.90) was significant. The parameter *ω* was smaller in the zero overlap (*ω* = 0.620 *±* 0.012) than in the full feature overlap condition (*ω* = 0.645 *±* 0.008). However, the main effect of response (*F*(1,27) = .180, *p* = .675, *η*_p_^2^ = .007, 90% CI [.000; .121], BF_10_ = 3.03) was not significant. The feature overlap by response interaction was significant (*F*(1,27) = 9.618, *p* = .004, *η*_p_^2^ = .263, 90% CI [.054; .449], BF_10_ = 14.42). Further post hoc *t* tests showed that when response had to be switched, the parameter *ω* was smaller in the no feature overlap condition (*ω* = 0.600 *±* 0.088) as compared to the full feature overlap condition (*ω* = 0.661 *±* 0.041) (*t*(27) = −3.45; *p* = .001). In contrast, there was no significant difference between no feature overlap and full feature overlap conditions, when the response had to be repeated (*t*(27) = 0.81; *p* = .214).

## 4 DISCUSSION

In the current study, we performed an in‐depth analysis of neurophysiological processes underlying response selection mechanisms in the TEC framework. The goal of this study was to identify neurophysiological correlates of event file processing at multiple levels of inspection and to link cognitive‐theoretical propositions to neurophysiological correlates of response selection. To this end, we examined EEG data at the ERP level, applied a temporal EEG signal decomposition procedure, and examined theta frequency network organization.

The behavioral data show robust event file binding effects replicating various previous findings (e.g., Colzato, Raffone, & Hommel, 2006; Colzato, Warrens, & Hommel, 2006; Hommel, 1998; Petruo et al., 2016). The robustness of findings is supported by the observed effect size and the Bayes factor providing evidence for the interaction “feature overlap x response”. Whenever there was a strong overlap between features of the S1 and the S2 stimulus, responses accuracy decreased when the response had to be changed. This reflects partial repetition costs because the previously established stimulus–response bindings and expectancies on stimulus–response associations are only partially fulfilled (Colzato, Warrens, & Hommel, 2006; Hommel, 2004). In contrast, the strong overlap between S1 and S2 features improved response accuracy when responses were repeated. This is in line with previous behavioral studies using this task (Colzato, Warrens, & Hommel, 2006; Hommel, 2004; Hommel, 2005; Petruo et al., 2016). Furthermore, the behavioral analyses provided evidence that event file coding occurs without a voluntary evaluation of S1 from the participants [for this method, see Colzato, Raffone, & Hommel, 2006; Petruo et al., 2016]. Thus, our results are in line with previous studies showing that attention to the first stimulus is not required for initial binding after the presentation of S1 (Hommel, 1998; Hommel, 2004; Kühn et al., 2011). On the neurophysiological level, and in line with the hypotheses, reliable binding processes (i.e., interactions “feature overlap × response”) were evident for the P3 ERP‐component amplitudes and for the C‐cluster amplitudes in the P3 time window. Moreover, the network organization of theta activity (and partially, alpha activity) quantified using the small‐world network metric revealed robust binding effects. Below, we will first discuss the time domain results and then network domain results. Finally, we compare the possible advantages of studying binding processes from the time domain perspective and from a small‐world network perspective.

### 4.1 Time domain findings

Regarding the P3 amplitudes and the C‐cluster amplitudes in the P3 time window, the results revealed an interaction “feature overlap × response.” This interaction was slightly stronger for the C cluster than for the P3 ERP component, as indicated by the effect sizes in the interaction effect, and the confidence intervals for the effect sizes. Effect sizes for the C‐cluster interaction effect, surpassed the criterion of the sensitivity analysis, which suggests that the observed effects are reliable. Furthermore, the Bayes factor of the interaction in the C‐cluster was high (BF > 10), thus provides strong evidence for the interaction “feature overlap × response” and corroborates the robustness of the finding. The reason is that the applied decomposition method (RIDE) uses an L1‐norm estimation to decompose ERPs, which reduces intraindividual variability. Standard ERPs are based on averaging and minimize the L2 norm of the data, which is more sensitive to intraindividual variability (Ouyang et al., 2011; Ouyang et al., 2015; Ouyang et al., 2017). Therefore, the relatively low trial numbers used for the ERP analysis could be accounted for higher intraindividual variability, which affected the RIDE analysis in lesser extent. Interestingly, the S‐cluster and the R‐cluster did not reveal effects of experimental manipulations. This is corroborated by a Bayesian analysis of the data and in line with the hypotheses. This lack of effects in the S‐ and R‐cluster suggests that purely stimulus‐related processes (like perception and attention) and purely response‐related processes (like motor preparation/execution) (Ouyang et al., 2011; Ouyang et al., 2015; Ouyang et al., 2017) do not capture the dynamics of processes occurring in an event file. This is completely in line with the TEC framework stating that it is the binding/association between stimulus features and response features that are accomplished in an event file. Although object files and action files are part of the event file (Hommel, 2009), the structure of the object and the action file itself is not changed during event binding processes (Hommel, 2004). Exactly this is suggested by the lack of interaction for the S‐cluster and the R‐cluster data. Although the applied temporal decomposition method was originally developed to account for intraindividual variability in EEG data (Ouyang et al., 2011; Ouyang et al., 2015; Ouyang et al., 2017), it has been shown that it can be used to dissociate different coding levels in a theoretically meaningful way in EEG data (Mückschel, Chmielewski, et al., 2017; Mückschel, Dippel, & Beste, 2017). In line with these hypotheses, the results suggest that a temporal EEG signal decomposition procedure reveals a result that well reflects theoretical principles of TEC. It is likely that the S‐cluster reflects object file related processes and the R‐cluster action file related processes. This may further be tested using experiments specifically measuring object and action file processing.

According to TEC, event file processes are concerned with the binding of stimulus features to response features. Several lines of evidence suggest that particularly the C‐cluster may reflect stimulus–response translation processes (Ouyang et al., 2017; Verleger et al., 2014; Wolff, Mückschel, & Beste, 2017). The current results corroborate this using a stringent theoretical framework. In detail, the results show that amplitudes in the P3 ERP and the C‐cluster in the P3 time window are small if S1 and S2 stimuli share many features and if no change in responses was required. At the behavioral level, this condition was associated with better performance. Several lines of evidence show that the P3 becomes smaller when response selection becomes more difficult (Falkenstein et al., 1994; Twomey et al., 2015; Verleger et al., 2005), when more processing resources have to be allocated (Polich, 2007) and when responses have to be switched/alternated (Barceló, Muñoz‐Céspedes, Pozo, & Rubia, 2000; Gajewski & Falkenstein, 2011; Gajewski, Kleinsorge, & Falkenstein, 2010; Hsieh & Liu, 2009; Karayanidis, Coltheart, Michie, & Murphy, 2003; Kieffaber & Hetrick, 2005; Lorist et al., 2000; Rushworth, Passingham, & Nobre, 2002). A reduction of the amplitudes in the P3 and the C‐cluster indicates that the above‐mentioned processes will be intensified. However, these accounts would also suggest that the reduction of amplitudes takes place when the allocation of response selection capacities was successful. As we saw in the behavioral results, in conditions, where responses were repeated, performance was improved. In the condition, in which the response was alternated, no further modulation of the amplitude takes place. This indicates that with a combination of high feature overlap between S1 and S2 and a simultaneous alternation of the response, not enough response selection capacities can be mobilized. Consequently, the behavioral performance became worse. Alternatively, the P3 and the C cluster amplitudes could reflect the amount of reactivation or retrieval of the S–R links (Verleger et al., 2015; Verleger et al., 2017). In this case, a full feature overlap with response repetition would reflect a simple, nonconflicting reactivation, hence, a small P3 or C cluster amplitude. In contrast, a no overlap condition with response repetition would indicate a retrieval of the S–R link and the reconfiguration of it. Consequently, this condition was characterized by larger P3 and C cluster amplitude. Interestingly, response alternation conditions did not show amplitude modulation. While no feature overlap with response alternation does not require any retrieval, a full feature overlap would reactivate the original S–R link. Thus, it is possible that the P3 and C cluster modulations observed after the presentation of S2 reflect the success of response selection capacities (Falkenstein et al., 1994; Twomey et al., 2015; Verleger et al., 2005), rather than the retrieval or reactivation of S–R links (Verleger et al., 2015; Verleger et al., 2017). The source localization using sLORETA suggests that regions in superior frontal gyrus (BA6), the medial frontal cortex (BA24), anterior cingulate cortex (BA24) and the inferior parietal cortex (BA40) were modulated by interactive effects between “feature overlap × response” for the P3 and the C‐cluster. These regions have previously been suggested to be involved in event coding processes (Chmielewski & Beste, 2019a; Chmielewski & Beste, 2019b; Chmielewski & Beste, 2019c; Elsner et al., 2002; Kühn et al., 2011; Petruo et al., 2016; Petruo et al., 2018; Zmigrod et al., 2014). However, the source localization findings were most consistent for the inferior parietal cortex (BA40), since this area was seen both in the sLORETA using the C‐cluster data and the nondecomposed P3 ERP data. Inferior parietal regions have previously been shown to be associated with modulations in the P3 (Verleger et al., 1994) and are central for response selection processes (Chersi, Ferrari, & Fogassi, 2011; Karch et al., 2010; Mückschel et al., 2014). A more overarching conceptual view on the function of inferior parietal regions suggests that this area is important to update internal representations using task‐relevant stimuli to initiate appropriate actions (Geng & Vossel, 2013). Exactly these aspects are at the core of event file binding processes (Hommel, 2009). A recent study supported this notion by showing that binding effects in event file related processes were shown in the C‐cluster and were related to activity in the BA40 (Opitz et al., 2020).

### 4.2 Network findings

In the time–frequency domain, we expected that theta and potentially alpha oscillations have a role in binding processes (Cavanagh & Frank, 2014; Clarke et al., 2018; Cohen, 2014; Crivelli‐Decker et al., 2018; Tóth et al., 2017). In terms of the network architecture, and using the small‐world metric, the results show a clear interaction “feature overlap × response” that directly reflects the interaction observed at the behavioral level. The obtained effect size in the interaction (i.e., eta squares) was larger for the theta frequency small‐world metric, compared to the parameters in the time domain analysis (cf. C‐cluster and P3 data, see also the related confidence intervals for effect sizes). The interaction is also supported by the Bayesian analysis. This suggests that network measures may be especially suitable to describe the neurophysiological dynamics of event file binding processes.

Regarding the small‐world network perspective, partial repetition costs at the behavioral level were paralleled by a larger (more positive) small‐world network parameter. Opposed to this, partial repetition benefits occurring when the response was not changed, when there was a strong overlap between S1 and S2 features, were reflected by a smaller small‐world network parameter. A small‐world‐like network architecture enables an efficient separation and functional integration of information (Achard & Bullmore, 2007; Bassett & Bullmore, 2006; Bullmore & Sporns, 2009). According to Telesford et al. (2011), a more random network organization (indicated by larger small‐world values) is less efficient. Previous results have already shown that network organization in the theta band becomes more random (inefficient) when demands on response selection processes increase (Beste et al., 2019). That data also suggest that the theta network architecture is more important to consider than pure theta power aspects when it comes to response selection processes (Beste et al., 2019). That is, information processing efficiency in the network plays a more important role in event‐file coding than the power of the neuronal activity (theta power results of the current study are available in the Supplementary materials). The current results suggest that whenever event file binding leads to partial repetition costs and complicates response selection, the associated theta network architecture is less small‐world like (i.e., more random) and hence less efficient. Whenever event file binding is associated with partial repetition benefits and increases response selection accuracy, the associated theta network architecture is more small‐world like and hence more efficient. A small‐world architecture has been suggested to be very efficient because this network architecture shows dense local interconnectivity and short average path length, thus linking nodes in a short and efficient way (Achard & Bullmore, 2007; Bassett & Bullmore, 2006; Bullmore & Sporns, 2009). From a biophysical point of view, it is reasonable that theta networks are important during event file processes (Cavanagh & Frank, 2014; Clarke et al., 2018; Cohen, 2014; Crivelli‐Decker et al., 2018; Tóth et al., 2017). Imaging studies suggest that event file processes depend on a widely distributed brain areas (Chmielewski & Beste, 2019a; Chmielewski & Beste, 2019b; Chmielewski & Beste, 2019c; Elsner et al., 2002; Kühn et al., 2011; Petruo et al., 2016; Petruo et al., 2018; Zmigrod et al., 2014). Also, the cognitive conception of event files, according to which the association of stimulus and response‐related processes is at stake (Hommel, 2004), implies that widely distributed areas from sensory processing to motor implementation are relevant. For information processing between such distant neural assemblies especially low‐frequency, high‐amplitude oscillations are suitable to integrate information across spatial distances (Buzsáki & Draguhn, 2004). Importantly, apart from the theta band, delta and alpha could be also potential candidates as they are characterized by low frequencies and high amplitudes. In the current study, we analyzed small‐world network activity in the alpha band, as well. Interestingly, in the alpha band, “feature overlap × response” interaction occurred only with one threshold parameter (85%), which showed difference between the no feature overlap with response alternation and the full feature overlap with response alternation conditions. Similar to the theta band results, the latter one was characterized by a more random (less effective) network architecture. However, unlike in the theta network, response repetition conditions did not differ from each other. Moreover, with a more conservative threshold (90%), the “feature overlap × response” interaction was not significant. Thus, network dynamics in the theta band show robust binding effects akin to the behavioral results. At the same time, the alpha network shows only partial sensitivity to the binding processes, and it is more sensitive to the overall strength of the network. It is possible, that the alpha network is related to general response selection processes (Clarke et al., 2018; Crivelli‐Decker et al., 2018; Tóth et al., 2017) rather than event file coding. In conclusion, the binding processes including retrieval, unbinding, and rebinding of S–R links are primarily reflected by the theta network. Nevertheless, future studies warranted to specifically investigate the potential role of network activities in the delta band. Intriguingly, and according to the TEC framework, processes in the event file can be understood in terms of network processes (Hommel, 2005; Hommel, 2009) in that an event file resembles a network of stimulus and response feature bindings (Hommel, 2009). Partial repetition costs and partial repetition benefits have been suggested to reflect a direct consequence of the network‐like processes in event files, that is, integration of information during event file retrieval, unbinding, and reconfiguration. The current results attribute this network‐like dynamic during event file processing to mechanisms in the theta frequency band and suggest that the small‐world network metric is suitable to describe network dynamics occurring in event files on a neurophysiological level. At present, the TEC framework does not yet contain a statement about the exact organization of (neurophysiological) network aspects during event file coding. Therefore, the analysis of neurophysiological data from the perspective of network organization contributes to the further development of the TEC framework.

### 4.3 Comparison of the time domain and network findings

The current study aimed to provide an in‐depth analysis of the neurophysiological underpinnings of event file binding processes. To gain reliable effects, we investigated the possible neural effects similar to the well‐known behavioral phenomena: we analyzed partial repetition benefit and partial repetition cost with the tools of ERP, signal decomposition (RIDE), and small‐world network analyses. Importantly, these methods have complimentary roles: they represent different temporal and spatial perspectives, specificities, and sensitivities to intraindividual variability. Thus, the results presented in the current study are hardly comparable directly to each other. Specifically, results from the time domain analyses represent focal results from a single electrode. In contrast, the small‐world network metric takes into account all possible electrode pairs. Additionally, the time domain results reflect the combination of different frequency bands, while the network analyses are specific for the theta and alpha oscillations. Finally, the RIDE decomposition provided clearer results in the C cluster compared to traditional ERP analysis. Unfortunately, this process was not available for the network analysis; therefore, the small‐world networks reflect nondecomposed activities. RIDE includes an iterative realignment of the single‐trial EEG data, separately for each electrode to reduce intraindividual variability (Ouyang et al., 2013; Ouyang et al., 2015). As a result, RIDE can distort the power and phase relationships of EEG data. Since these information are crucial to calculate the coherence between electrodes, signal decomposition would lead to nonreliable small‐world metric analyses. In conclusion, both time domain and network‐based approaches represent potential advantages to understand the neurophysiology of event file binding. The parallel use of methods has important theoretical implications: The different sensitivities allow us to capture event file processes both at the local (subprocess), and global (information processing efficiency) levels. In the current study, modulation of the P3 and the C cluster amplitude likely reflected the allocation of response selection resources during event file coding. This is a core aspect of event file coding; however, it is unlikely to reflect all subprocesses related to binding, retrieval, unbinding, and reconfiguration. At the same time, the small‐world network characteristics predominantly in the theta band reflected the separation and integration of information needed to successfully solve the task. Importantly, while a subprocess can be tied to a certain area, the global perspective reflected by the small‐world analysis represent a more widely distributed network. Please, note, that while in the source localization analyses BA40 proved to be the most consistent source of activation across the P3 and C‐cluster effects, in both cases, other areas were implicated too. That is, even from a more focal, subprocess‐based perspective binding effects do not resemble single source activation. In sum, from different lenses, both the localized and the distributed effects present valid neurophysiological underpinnings for event file processes.

### 4.4 Conclusions

In summary, we linked EEG signal decomposition methods to theoretical components of the TEC framework to delineate the neurophysiological mechanisms underlying event file binding, including the retrieval and reconfiguration of event files. In particular, we show that event file binding is associated with modulations in the P3 ERP component and is associated with processes in the inferior parietal cortex (BA40). However, ERPs reflect a mixture of processes. From the perspective of TEC, it is important to dissociate binding processes occurring in an event file from stimulus‐related and response‐related processes in the object file and the action file. We show that a temporal EEG signal decomposition reveals a pattern of results suggesting that event file processes can be isolated using signal decomposition. The decomposition result is in line with the theoretical assumption that event files mediate stimulus–response association/binding processes. Most important, however, is the finding that event file binding processes are strongly reflected by modulations in the organization of networks in the theta frequency band using a small‐world metric. The effects of partial repetition cost and benefit are associated with modulations of the network towards an inefficient or more efficient organization of the network, respectively. In this respect, neurophysiological network measures correspond to the processes that have already been assumed on the cognitive‐theoretical level. Taken together, theoretical propositions of TEC can be translated into human neurophysiology.

## Supporting information


**Appendix** **S1**. Supporting Information

## Data Availability

The data that support the findings of this study are available from the corresponding author upon reasonable request.
