# Supplementary material for: Connecting EEG signal decomposition and response selection processes using the theory of event coding framework
Source: Hum Brain Mapp. 2020 Mar 9;41(10):2862–77. doi: 10.1002/hbm.24983 (PMC7294061; doi:10.1002/hbm.24983)
Supplement: Supplementary file 1 — Appendix S1. Supporting Information [file HBM-41-2862-s001.docx]

**Supplemental Material**

**Linking EEG signal decomposition and response selection processes using the Theory of Event Coding framework**

Adam Takacs, Nicolas Zink, Nicole Wolff, Alexander Münchau, Moritz Mückschel, Christian Beste


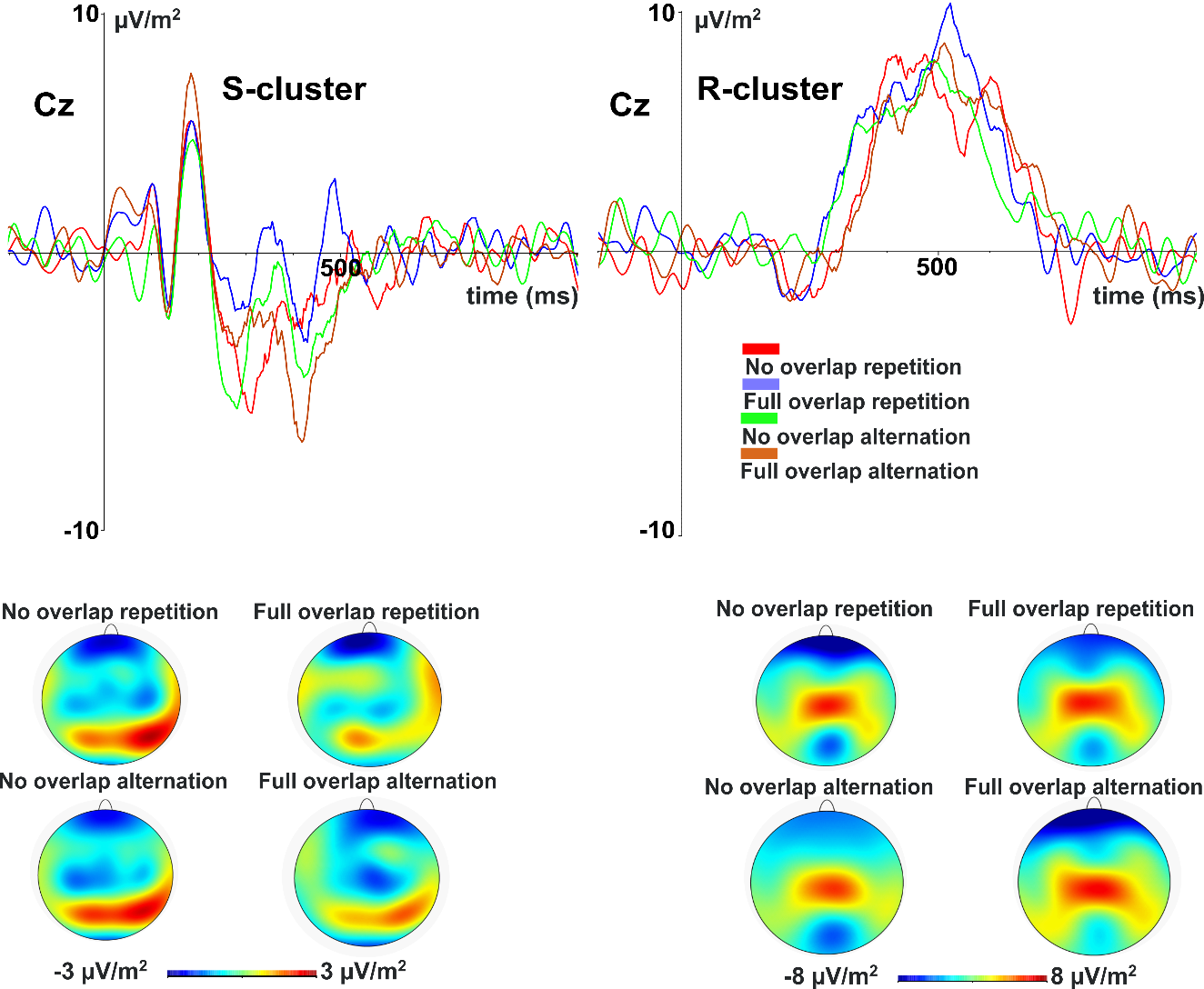


***Supplementary Figure 1.*** *S-cluster (on the left) and R-cluster (on the right) results at electrode Cz. Stimulus-locked activities are shown across four conditions: no compatibility repetition (red), full compatibility repetition (blue), no compatibility alternation (green), and full compatibility alternation (brown). Time point zero denotes the stimulus presentation. The scalp topography plots show the distribution of the mean activity across the four conditions for the time window 400 ms – 700 ms. For related analyses, please, see 3.2.1. Time domain level analyses, in the main text.*

The S-cluster data was analysed for stimulus-related processes, namely for the P1 and N1 components. S-cluster data for the analysed channels (P9 and P10) is presented in Supplementary Figure 2.

To analyse the stimulus-locked P1 component, we selected the electrode P10 with a time window of 80 to 130 ms, based on scalp topography. Within this time interval, the mean amplitude was quantified and extracted at the single subject level. The compatibility by response ANOVA on the mean amplitude of the S-cluster P1 showed that the main effects of compatibility (*F*(1,27) = 1.12, *p* = .299, η_p_^2^ = .040), and response type (*F*(1,27) = .24, *p* = .627, η_p_^2^ = .009) were not significant. Similarly, the compatibility by response interaction was not significant (*F*(1,27) = .86, *p* = .362, η_p_^2^ = .031, *BF*_10_ = 0.06). The Bayes factor supports the H_0_ over the H_1_. To analyse the stimulus-locked N1 component, we selected the electrode P9 with a time window of 140 to 190 ms, based on scalp topography. Within this time interval, the mean amplitude was quantified and extracted at the single subject level. The compatibility by response ANOVA on the mean amplitude of the S-cluster N1 showed that the main effects of compatibility (*F*(1,27) = 2.77, *p* = .108, η_p_^2^ = .093), and response type (*F*(1,27) = .84, *p* = .367, η_p_^2^ = .030) were not significant. Similarly, the compatibility by response interaction was not significant (*F*(1,27) = .05, *p* = .833, η_p_^2^ = .002, *BF*_10_ = 0.17). The Bayes factor supports the H_0_ over the H_1_.


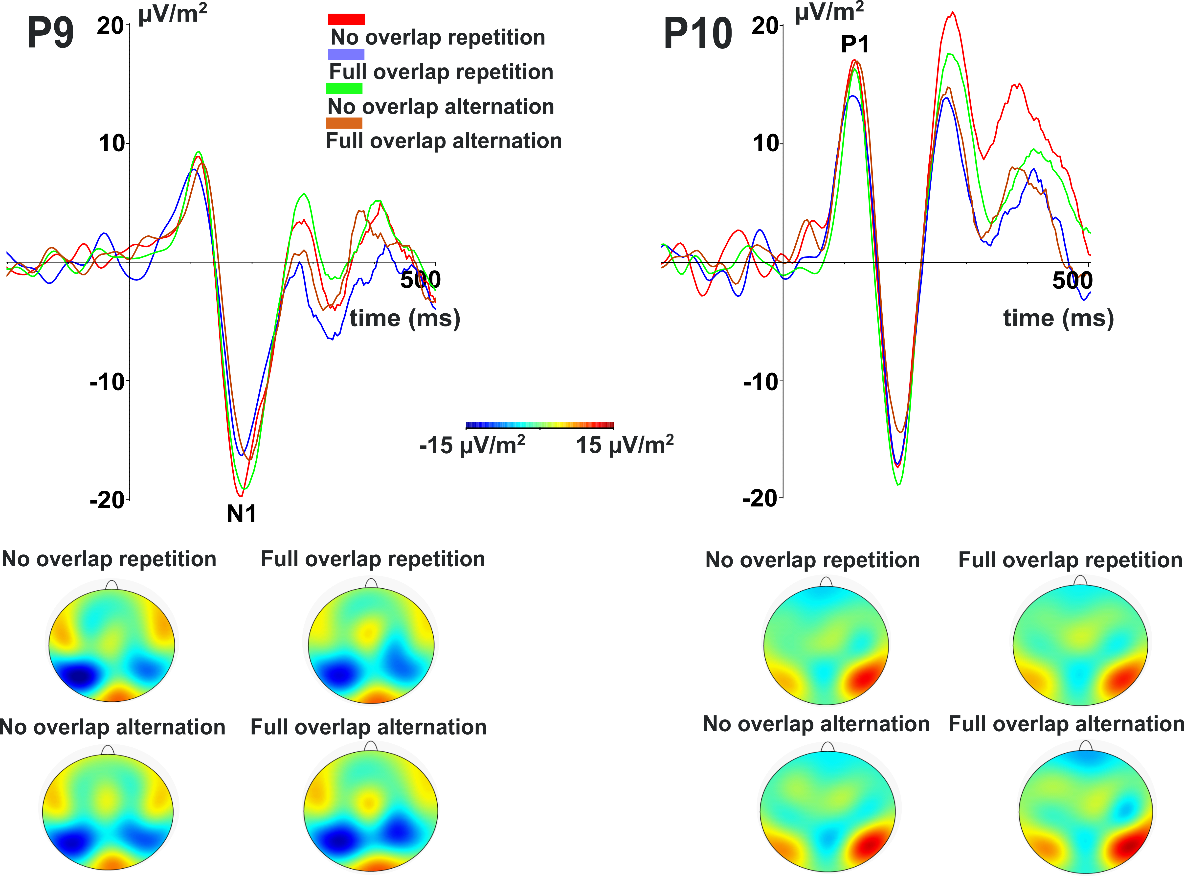


***Supplementary Figure 2.*** *S-cluster results at electrodes P9 and P10. The S-cluster P1 and N1 components are shown across four conditions: no compatibility repetition (red), full compatibility repetition (blue), no compatibility alternation (green), and full compatibility alternation (brown). Time point zero denotes the stimulus presentation. The scalp topography plots show the distribution of the mean activity across the four conditions for* *the time windows of the P1 and N1.*

*Time-Frequency decomposition*

Calculations of the time-frequency decomposition and the small-world metric (see below) were carried out only for the non-decomposed EEG data and not when RIDE decomposition had been performed beforehand. The reason is that the RIDE procedure includes an iterative realignment of the single-trial EEG data to reduce intra-individual variability in the data [Ouyang et al., 2013; Ouyang et al., 2015]. This is done for each electrode separately (i.e. without accounting for the relative phase of the signal between the electrodes) [Ouyang et al., 2013; Ouyang et al., 2015]. Therefore, RIDE can distort the power and phase-relationships of EEG data. Since especially the latter is central to calculate the coherence between electrode as basis for further small-world metric analyses, TF RIDE decomposed data was not used for time-frequency and small-world network calculations.

Time-frequency (TF) decomposition was run on the single-trial time-domain using Morlet wavelets (*w*, Morlet parameter c = 5.5) in the frequency range from 1 to 20 Hz, with frequency steps of 1 Hz:


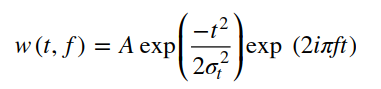


Where t is time, *A* = (σ*_t_*√π)^-1/2^, σ*_t_* is the wavelet duration, and *i* = √-1. We used a ratio of *f*_0_/σ*_f_* where *f*_0_ is the central frequency and σ*_f_* is the width of the Gaussian shape in the frequency domain. The time-frequency decomposition was computed on single-trial data for each of the segmented conditions to calculate the total wavelet power. After TF-decomposition, the data were averaged at the single-subject level to analyze the power of the theta frequency oscillations. For statistical analysis, the maximum TF total power was quantified in the theta frequency band as a complex value, and it was log-transformed to normalize the distributions. First, the power of the theta frequency oscillations was analyzed between 4 Hz and 8 Hz (*f*_0_ = 6 Hz) and quantified at electrode Cz, in line with the quantification of the ERPs. Mean theta frequency power was analyzed from the onset of S2 to 850 ms afterward. An 850 ms long time window is sufficient enough to include at least one cycle of the lowest frequency, and seven cycles of the highest frequency of the theta band oscillations. Increasing this time window would have captured a larger amount of variance of the theta activity, however, it would also potentially led to overlapping segments. In additional analyses also other electrode sites were chosen (cf. results section for details).

*Analysis of frequency power data*

The theta power data were analyzed in two-way repeated measures ANOVA with feature overlap (full vs. no feature overlap) and response (repetition vs. switch) as within-subject factors. The time-frequency plots showing alpha and theta band power as a complex value are given in Supplementary Figure 3 for each condition.

The feature overlap by response ANOVA on the complex value of theta power at electrode Cz showed that the main effects of feature overlap were significant (*F*(1,27) = 4.85, *p* = .04, η_p_^2^ = .152). Theta power was larger at the full feature overlap (1.80 *±* 0.37) than at the null feature overlap condition (1.74 *±* 0.38). The main effect of response was not significant (*F*(1,27) = 0.48, *p* = .496, η_p_^2^ = .017). Similarly, the interaction between feature overlap and response type was not significant (*F*(1,27) = 2.71, *p* = .111, η_p_^2^ = .091, *BF*_10_ = 0.066). The Bayes factor for the feature overlap by response interaction supports the H_0_ over the H_1._ Further analyses using parietal electrode sites and all parietal electrode sites combined into one cluster again revealed no interaction between feature overlap and response type was not significant (all *F* < 1.55, *p* > .311, *BF*_10_ = 0.045). Therefore, power of theta frequency activity at various electrode sites did not show binding effects as reflected by the behavioral data.


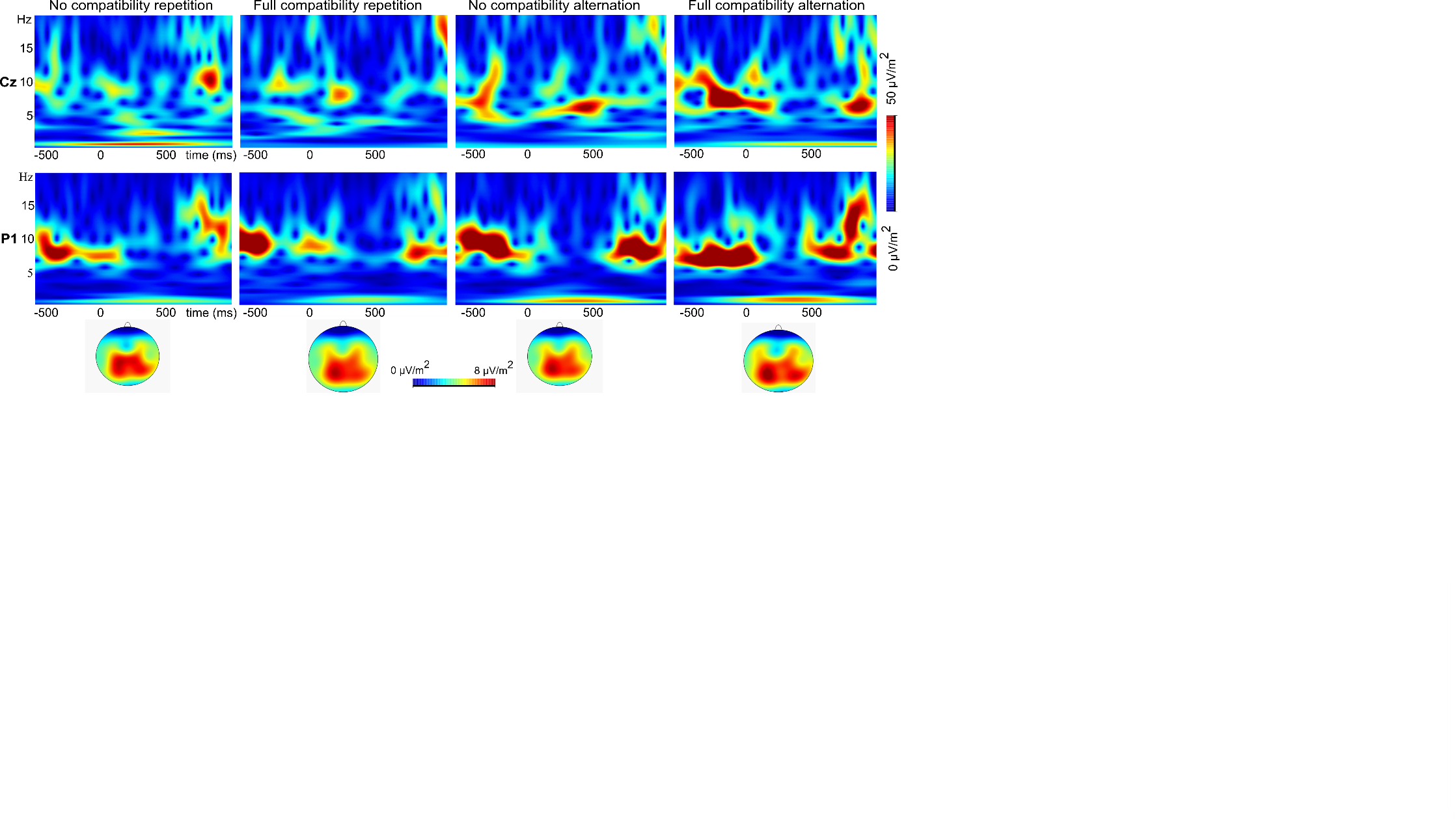


***Supplementary Figure 3.*** *Time frequency decomposition results. The top row shows the time-frequency decomposition data across the four conditions at the Cz electrode: no compatibility repetition, full compatibility repetition, no compatibility alternation, full compatibility alternation. The second row shows the time-frequency decomposition data across the experimental conditions at the P1 electrode. The scalp topography plots are given for the 850 ms time period after the stimulus presentation.*
